# Infants' Food

**Published:** 1891-01-31

**Authors:** 


### EDITOR'S LETTER-BOX.

[Our Correspondents are reminded that prolixity is a great bar to publication, and that brevity of style and concieness of statement greatly facilitate early insertion.]

### INFANTS' FOODS.

Messrs. J. R. Neave and Co. write: Our attention has been called to a comparison of "Neave's Food" with "Nestlé's Milk Food," which has appeared in your journal, under the heading of "Infants' Foods," and as such a comparison is calculated to mislead, we beg leave to point out that our Infants' Food being a pure cereal preparation, as is stated on the tins, contains, therefore, neither milk nor sugar, which require to be added when it is prepared for use, which, we consider, is a decidedly preferable form to a combination of these with wheaten flour, as is the case with Nestlé's Food, which, according to their own statement, is prepared from milk, wheaten flour, and sugar combined. In order, therefore, to make a fair comparison of these two Foods, an analysis of our own should be made when it is prepared for use with the addition of milk and a little sugar, which has been most carefully done by Dr. A. Stutzer, of Bonn, Director of the Chemical Laboratory of Rhenish Prussia, with the following result: Fat, 9·10; nitrogenous matter (albumenoids), 18·12; cellulose, 1·85; carbo-hydrates, 75·03; salts, 2·89. And in comparing our food with human milk, Dr. Stutzer has stated that, "as regards the proportion of flesh-forming albuminoids and the bone-forming salts, there exists a perfect uniformity in connection with these two Foods." Other extracts from the testimonials of well-known English and German doctors could be also given in confirmation of the above. We, therefore, feel justified in saying that our Food, prepared according to the directions given, is as good a substitute for mother's milk as can be obtained, and this is abundantly confirmed by many unsolicited testimonials from those who have used and proved the value of the Food.

